# Dog Bites

**Published:** 1884-07

**Authors:** 


### DOG BITES.

Mr. T. M. Dolan, F. R. C. S., the author of a standard work on hydrophobia, has drawm up the folowing simple remedies for the immediate treatment of dog-bites: "The bite of a healthy dog cannot cause hydrophobia. This is a well established fact. As it is difficult to determine the state of health of a dog at the time he bites, the wound should be treated as if the dog were rabid. Dog-bites should be treated at once by the person bitten or by the bystander, by sucking the wound if possible; enlarging the wound with a penknife to encourage bleeding; by hot water fomentation; by free washing with cold water; by ligature, a piece of string tied between wound and heart. After bleeding has been encouraged and the wound has been well washed apply hot iron—as a heated penknife, small key, &c.—caustics, pure nitric, sulphuric or hydrochloric acid, nitrate of silver, acetic acid, carbolic acid, ammonia, salt, Condy's fluid or piece of hot cinder. If near a chemist's the person bitten should run there, keeping his mouth applied to the wound, if possible, and spitting out the blood extracted. If near a medical man's house, run there at once. If in a part where the person bitten cannot apply his mouth, some bystander should suck the wound—no harm can follow from thus lending assistance. The dog inflicting the bite should be kept under observation for at least fourteen days. It will soon be seen whether it is healthy or not. If healthy, there is no fear of future development of hydrophobia. If the person bitten experiences shooting pain up the arms or other parts of the body, three or four Turkish baths should be taken. If the person bitten is nervous, he should place himself under the care of his medical attendant. I have treated some hundreds of cases of dog bites from all parts of the country, and I am glad to say that those bitten have not experienced any after symptoms."

